# *Ganoderma lucidum* Polysaccharides Reduce Lipopolysaccharide-Induced Interleukin-1**β** Expression in Cultured Smooth Muscle Cells and in Thoracic Aortas in Mice

**DOI:** 10.1155/2014/305149

**Published:** 2014-03-04

**Authors:** Chan-Jung Liang, Chiang-Wen Lee, Hsin-Ching Sung, Yung-Hsiang Chen, Yao-Chang Chiang, Hsien-Yeh Hsu, Ying-Chin Tseng, Chi-Yuan Li, Shu-Huei Wang, Yuh-Lien Chen

**Affiliations:** ^1^Department of Anatomy and Cell Biology, College of Medicine, National Taiwan University, No. 1, Section 1, Ren-Ai Road, Taipei 100, Taiwan; ^2^Department of Nursing, Division of Basic Medical Sciences, Chiayi Campus, Research Center for Industry of Human Ecology, Chang Gung University of Science and Technology, Taoyuan 303, Taiwan; ^3^Department of Anatomy, College of Medicine, Chang Gung University, Taoyuan 333, Taiwan; ^4^Graduate Institute of Integrated Medicine, China Medical University, Taichung 404, Taiwan; ^5^Center for Drug Abuse and Addiction, China Medical University Hospital, Taichung 404, Taiwan; ^6^Institute of Biotechnology in Medicine, National Yang-Ming University, Taipei 112, Taiwan; ^7^Department of Obstetrics and Gynecology, Hsinchu Cathay General Hospital, Hsinchu 300, Taiwan; ^8^Institute of Clinical Medical Science, China Medical University, No. 91 Hsueh-Shih Road, Taichung 404, Taiwan

## Abstract

The expression of inflammatory cytokines on vascular walls is a critical event in vascular diseases and inflammation. The aim of the present study was to examine the effects of an extract of *Ganoderma lucidum* (Reishi) polysaccharides (EORPs), which is effective against immunological disorders, on interleukin- (IL-) 1**β** expression by human aortic smooth muscle cells (HASMCs) and the underlying mechanism. The lipopolysaccharide- (LPS-) induced IL-1**β** expression was significantly reduced when HASMCs were pretreated with EORP by Western blot and immunofluorescent staining. Pretreatment with 10 **μ**g/mL EORP decreased LPS-induced ERK, p38, JNK, and Akt phosphorylation. But the increase in IL-1**β** expression with LPS treatment was only inhibited by pretreatment with the ERK1/2 inhibitor, while the JNK and p38 inhibitors had no effect. In addition, EORP reduced the phosphorylation and nuclear translocation of nuclear factor- (NF-) **κ**B p65 in LPS-treated HASMCs. Furthermore, *in vivo*, IL-1**β** expression was strongly expressed in thoracic aortas in LPS-treated mice. Oral administration of EORP decreased IL-1**β** expression. The level of IL-1**β** expression in LPS-treated or in LPS/EORP-treated group was very low and was similar to that of the saline-treated group in toll-like receptor 4-deficient (TLR4^−/−^) mice. These findings suggest that EORP has the anti-inflammatory property and could prove useful in the prevention of vascular diseases and inflammatory responses.

## 1. Introduction

The inflammatory processes have a key role not only in the initiation and progression of atherosclerosis, but also in the stability of the established atherosclerotic plaques [1, 2]. Cytokines of the interleukin-1 (IL-1) family play a pivotal role in regulating the immunoinflammatory responses, and extensive studies have been performed to determine whether IL-1 modifies the inflammatory response [3, 4]. IL-1*β* upregulates a substantial increase in the expression of adhesion molecules by vascular smooth muscle cells (VSMCs) *in vitro*, and these factors promote monocyte recruitment and infiltration into the arterial wall [5, 6] and induce VSMC migration and proliferation [3, 7, 8]. *In vivo* studies have shown increased levels of IL-1*β* mRNA in human atherosclerotic lesions [9] and of IL-1*β* mRNA and protein in VSMCs of atherosclerotic arteries from non-human primates [10]. Although the relationship between IL-1*β* and cardiovascular disease has been extensively studied, IL-1*β* expression and its related mechanism in LPS-induced human aortic smooth muscle cells (HASMCs) and in inflammatory vascular walls have not been studied in detail. Moreover, reducing IL-1*β* expression might be a beneficial approach for inhibiting the development of vascular inflammation and atherosclerosis [11].

*Ganoderma lucidum* (*G. lucidum*, Reishi), a popular home remedy, has long been known for its beneficial effects on human health and longevity and is used to treat chronic hepatopathy, hypertension, hyperglycemia, and neoplasia [12, 13]. *Ganoderma lucidum* intake caused an increase in plasma antioxidant capacity, and that 10 d supplementation was associated with a trend towards a decrease of plasma lipids in human subjects [14, 15]. Studies on the role of *G. lucidum* in regulating various body functions have revealed that *G. lucidum* polysaccharides are the bioactive constituents responsible for many of their health benefits, such as its antioxidant, anticancer, anti-inflammatory, and immunomodulatory activities [16–18]. The effects of* G. lucidum *on the immune system have been linked to the regulation of cytokine expression and differentiation of macrophages [19, 20] and the maturation of cultured murine bone marrow-derived dendritic cells [21]. *G. lucidum* have also been shown to suppress TNF-*α* expression from lipopolysaccharide- (LPS-) stimulated murine RAW 264.7 cells [22], reduce IL-1*β* secretion from activated microglia after LPS treatment [23], and prevent albumin-induced oxidative damage and chemokines synthesis in human proximal tubular epithelial cells [24]. Regulation of inflammatory cytokine expression requires a complex array of intracellular signaling pathways involving mitogen-activated protein kinases (MAPKs) and transcriptional factors [25, 26]. Although these multiple signaling molecules have received considerable attention, little is known about the effects of an extract of Reishi polysaccharides (EORPs) on IL-1*β* expression and the mechanisms of these effects, and a better understanding of this might provide important insights into the prevention of cardiovascular diseases and inflammation. We were therefore interested in understanding the mechanism of action of EORP on human aortic smooth muscle cells (HASMCs) stimulated by inflammatory cytokines and whether it affects the IL-1*β* expression, an important event in vascular diseases and inflammation. In addition, we studied the effects of EORP on IL-1*β* expression in thoracic aortas in LPS-treated mice. Our results showed that EORP attenuated IL-1*β* expression both *in vitro* and *in vivo* and that this effect was mediated, at least in part, through inhibition of extracellular signal-regulated kinase (ERK) phosphorylation, nuclear factor NF-*κ*B activation, and TLR4 activation.

## 2. Materials and Methods

### 2.1. Preparation of EORP

A crude powder of *G. lucidum* prepared via alkaline extraction with 0.1 N NaOH, followed by neutralization and ethanol precipitation, was obtained from Pharmanex (Provo, UT). The biologically active compounds from Reishi are identified as the fucose-containing glycoprotein fraction and named EORPs. The molecular structure and the quality control of EORPs were described in detail in the previous reports [13, 19]. In brief, the carbohydrate composition analyses of crude water soluble Reishi extract indicated that glucose, mannose, and fucose exist as the major components. The crude extract contains 15.6% proteins. The saccharides contain either a polysaccharide backbone with *β*-1,3-linkages or a polymannose backbone with *α*-1,4-linkages, both with side chains of unknown structure. EORPs were prepared as GMP grade and carefully monitored to meet the Food and Drug Administration standard. The EORP is currently commercially available as the traditional Chinese medicine.

### 2.2. HASMC Cultures

HASMCs, purchased as cryopreserved tertiary cultures from Cascade Biologics (OR, USA), were grown in culture flasks in smooth muscle cell growth medium (M231, Cascade Biologics Inc.). The cells were used between passages 4 and 8. All cells were synchronized in serum-free medium for 24 h prior to experiments. For all data shown, the experiments were repeated at least 3 times in duplicate with different preparations of HASMCs. For Western blot analysis, the graphical analysis represents the results from three independent experiments and quantification by densitometry.

### 2.3. Effect of LPS and EORP on Cell Viability

The cell viability was determined by 3-(4,5-dimethylthiazol-2-yl)-2,5-diphenyltetrazolium bromide (MTT) (Sigma-Aldrich, St. Louis, MO, USA) assay. HASMCs were plated in 96-well plates (1 × 10^4^ cells/well). After serum starvation, the cells were treated with different concentrations of LPS or EORP for 24 h, and then MTT solution was added to each well and incubated for another 2 h at 37°C in 5% CO_2_. The supernatant was discarded and dimethyl sulfoxide (DMSO) was added to dissolve the formazan crystals. The cellular viability was measured by ELISA plate reader at 540 nm.

### 2.4. Western Blot Analysis

Western blot analyses were performed as described previously [27]. In brief, samples of cell lysate (20 **μ**g of protein) were subjected to 12% SDS-PAGE and transferred to PVDF membranes, which were then treated with 5% nonfat milk in 0.1 M phosphate buffer for 1 h at RT to block nonspecific binding of antibody. To measure IL-1*β* levels, the membranes were then incubated with rabbit anti-human IL-1*β* (1 : 1000 dilution, GeneTex, Irvine, CA, USA) and then with horseradish peroxidase- (HRP-) conjugated goat anti-rabbit IgG antibodies (1 : 6000 dilution, KPL, Gaithersburg, MA, USA) and bound antibody was detected using Chemiluminescence Reagent Plus. In other studies, the antibodies used were rabbit anti-human phospho-Jun N-treminal kinase (JNK), rabbit anti-human phopho-ERK1/2, rabbit anti-human phospho-p38 (all 1 : 1000 dilution, Cell signaling, Danvers, MA, USA), rabbit anti-human phospho-p65, and rabbit anti-human p65 (all 1 : 1000 dilution, GeneTex) followed by HRP-conjugated goat anti-rabbit IgG antibodies. The intensity of each band was quantified using a densitometer. Antibodies against *β*-actin, *α*-tubulin, or GAPDH (all 1 : 1000 dilution, Gene Tex) were used as loading controls.

### 2.5. Immunocytochemical Staining

HASMCs were plated at a density of 10^4^ cells/well in gelatin-coated coverslips. After starvation, the cells were treated with various concentrations and time of LPS and EORP. After treatment, the cells were fixed in 4% paraformaldehyde for 15 min at room temperature and then incubated with IL-1*β* and NF-*κ*B p65 antibodies at 4°C overnight. After incubation, the cells reacted with FITC-conjugated anti-rabbit IgG (both from Sigma) for 1 h at room temperature. DAPI stain was used as the nuclear counterstain and observation was conducted by fluorescence microscope.

### 2.6. IL-1*β* Enzyme-Linked Immunosorbent Assay (ELISA)

The concentrations of IL-1*β* in cultured media of smooth muscle cell and in plasma from mice were determined using the human and mouse IL-1*β* ELISA kits (R&D Systems, Minneapolis, MN), respectively. The analysis was performed according to the instructions from the manufacturer.

### 2.7. Knockdown of Gene Expression Using Small Interfering RNA

Knockdown of ERK gene expression was performed by transfection with small interfering RNA (siRNA, Invitrogen, Carlsbad, CA, USA). HASMCs (10^6^) were resuspended in 100 **μ**L of Nucleofector solution (Amaxa Biosystems, Germany), and gene-specific ERK siRNA oligomers (1 **μ**M) were electroporated according to the manufacturer's instruction manual. The ERK siRNAs (catalog number 10620319 124945 F11 and 10620318 124945 F12, Invitrogen) were AUA UUC UGU CAG GAA CCC UGU GUG A and UCA CAC AGG GUU CCU GAC AGA AUA U. Cells were transfected for 48 h. The siRNA results were evaluated by Western blotting.

### 2.8. RNA Extraction and Reverse Transcription-Polymerase Chain Reaction (RT-PCR) Assay

Total RNA was extracted from cells using Trizol reagent (Invitrogen) according to the manufacturer's protocol. The reverse-transcriptase reaction was carried out using M-MLV reverse transcriptase (Invitrogen). Complementary DNA was generated by addition of total RNA (1 **μ**g) to a reaction mixture containing 0.5 **μ**g/**μ**L oligo-deoxythymidine, 20 mM dNTP, 0.1 M dithiothreitol, 250 mM Tris-HCl, pH 8.3, 375 mM KCl, and 15 mM MgCl_2_ and reaction at 37°C for 90 min. The oligonucleotide primers used were 5′-CAGACCATGATCACACAGGG-3′ (forward) and 5′-TGGAAAGATGGGCCTGTTAG-3′ (reverse) for ERK and 5′-GTAACCCGTTGAACCCCATT-3′ (forward) and 5′-CCATCCAATCGGTAGTAGCG-3′ (reverse) for 18 S subunit ribosomal RNA. The PCR conditions were an initial denaturation at 94°C for 5 min, followed by 29 cycles of denaturation at 94°C for 1 min, primer annealing at 55°C for 1 min, and extension at 72°C for 5 min. The products of PCR were analyzed by electrophoresis on 2% agarose gels stained with ethidium bromide.

### 2.9. Mouse Model

All animal experiments were carried out in accordance with the recommendations in the Guide for the Care and Use of Laboratory Animals of the National Institutes of Health. The protocol was approved by the Institutional Animal care and Use Committee of the National Taiwan University (Permit number: 20110011). Eight-week-old male C57BL6J mice and toll-like receptor 4-deficient (TLR4^−/−^) mice, weighing between 25 and 35 g, were used in the present study. To examine the effects of EORP administration on thoracic aortas in the LPS-treated mice, the C57BL6J mice were divided into two divisions. One division is defined as the acute inflammatory phase (2 days) and the other one is the chronic inflammatory phase (2 weeks). Each division was further divided into four groups. Groups 1 and 2 were given, respectively, either saline (control) or 1 mg/kg/day of LPS on day 1 or on days 1–13 by intraperitoneal (ip) injection, and group 3 was treated with LPS in the same way as group 2 but received oral EORP at 60 mg/kg/day on days 0-1 (acute inflammatory phase) or on days 0–13 (chronic inflammatory phase), and group 4 received only the oral EORP. Furthermore, the TLR4^−/−^ mice were divided into 3 groups; groups 1–3 were treated the same way as groups 1–3 of the chronic inflammatory division. The selection of EORP dose was based on the previous reports [17, 27]. At the end of the 2 days or 2 weeks of the experiments, the animals were anesthetized by ip injection of 60 mg/kg sodium pentobarbital and sacrificed. Blood samples were collected, and soluble IL-1*β* in the plasma was measured by ELISA. One segment of the thoracic aortas was rinsed with ice-cold PBS, immersion-fixed with 4% buffered paraformaldehyde and paraffin-embedded, and then cross-sectioned for immunohistochemistry, while the remaining portion was immediately frozen in liquid nitrogen for protein extraction. The tissue sections (5-6 **μ**m thick) were mounted on poly-l-lysine coated slides, deparaffinized, rehydrated, and washed with PBS. To study cellular expression and localization of IL-1*β*, serial sections were incubated with 1% hydrogen peroxide in methanol for 10 min to block endogenous peroxidase activity and to permeabilize the cells; then nonspecific binding was blocked by incubation for 1 h at RT with PBS containing 5 mg/mL of bovine serum albumin. In the primary antibody step at 37°C for 1 h, the first serial section was incubated with goat anti-human CD31 antibody (EC marker, 1 : 100, Santa cruz, CA, USA), the second with goat anti-human IL-1*β* antibody (1 : 50), and the third with mouse anti-*α*-smooth muscle actin (SMC marker, 1 : 50, Dako Cytomation, CA, USA). The first and second slides were localized by an indirect immunoperoxidase technique (avidin-biotin-horseradish peroxidase complex) employing diaminobenzidine (Vector) as chromogen, while bound antibodies on the third section were then incubated for 1.5 h at RT with FITC-conjugated goat anti-mouse secondary antibody (1 : 400, Sigma Chemical Co., St. Louis, MO, USA) and observed by fluorescent microscopy. Each incubation was followed by three times of 5 min washes in PBS. Negative controls were performed by omitting the primary antibody.

### 2.10. Statistical Analysis

Values are expressed as the mean ± SEM. Student's *t*-test and one-way ANOVA were performed to compare the two groups and multiple groups, respectively. A *P* value <0.05 was considered statistically significant.

## 3. Result

### 3.1. EORP Reduces IL-1*β* Expression in LPS-Treated HASMCs

When the cytotoxicity of LPS or EORP for HASMCs was detected by the MTT assay after 24 h of incubation, cell viability was not affected by the presence of 1–15 **μ**g/mL of LPS or 1–10 **μ**g/mL of EORP (data no shown).

**Figure 1:**
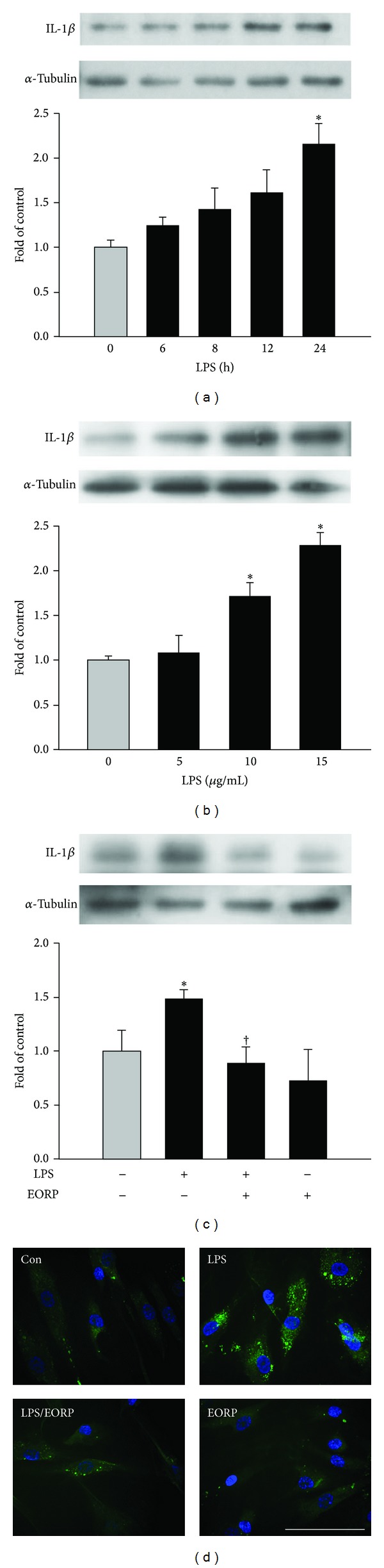
EORP reduces IL-1*β* protein expression in LPS-treated HASMCs. (a) and (b) HASMCs were treated with 10 **μ**g/mL of LPS for the indicated time (a) or with the indicated concentration of LPS for 24 h (b). (c) HASMCs were incubated for 24 h with 10 **μ**g/mL of EORP and then for 24 h with 10 **μ**g/mL of LPS in the continued presence of the same concentration of EORP, and then IL-1*β* expression was measured in cell lysates by Western blotting. *α*-Tubulin was used as the loading control. (d) The cells were treated as in panel (c), and then the distribution of IL-1*β* was analyzed by immunofluorescent microscopy. IL-1*β* expression is indicated by green fluorescence (FITC) and nuclei by blue fluorescence (DAPI). Bar = 100 **μ**m. In panels (a), (b), and (c), the data are expressed as a fold value compared to the control value and are the means ± SEMs (*n* = 3). *P < 0.05 as compared to the untreated (control) cells. ^†^ *P* < 0.05 as compared to the LPS-treated cells.

To determine the optimal conditions for LPS-induced IL-1*β* expression by HASMCs, we first performed dose-response studies in which HASMCs were cultured with various concentrations of LPS for various time intervals. As shown in Figure 1(a), IL-1*β* expression in HASMCs with 10 **μ**g/mL LPS treatment reached a maximum after 24 h (2.3 ± 0.2). IL-1*β* expression was induced in a dose-dependent manner after treatment with 5, 10, or 15 **μ**g/mL of LPS for 24 h (1.1 ± 0.2, 1.7 ± 0.1, 2.3 ± 0.1, resp., of control levels) (Figure 1(b)). The induction caused by the two highest concentrations was significant. As shown in Figure 1(c), when the HASMCs were pretreated with 10 **μ**g/mL of EORP for 24 h before incubation with 10 **μ**g/mL of LPS for 24 h, LPS-induced IL-1*β* expression was reduced to 0.9 ± 0.1 of control levels. The effect of EORP on IL-1*β* expression was also studied by immunofluorescence staining (Figure 1(d)). In untreated cells, IL-1*β* expression was weak. In contrast, cells treated for 24 h with LPS showed strong IL-1*β* expression and this effect was inhibited by pretreatment with EORP. Furthermore, unstimulated HASMCs produced low amounts of IL-1*β* in the cell culture supernatant (80 ± 23 pg/mL), and 24 h treatment with 10 **μ**g/mL of LPS resulted in a marked increase in IL-1*β* levels (653 ± 90 pg/mL) by ELISA. This effect was significantly inhibited by 24 h preincubation with 10 **μ**g/mL of EORP (398 ± 50 pg/mL). In all subsequent experiments, unless otherwise specified, 10 **μ**g/mL LPS and 10 **μ**g/mL EORP were used.

### 3.2. EORP Attenuated LPS-Induced IL-1*β* Expression is Partly Dependent on Inhibition of ERK1/2 Phosphorylation

**Figure 2:**
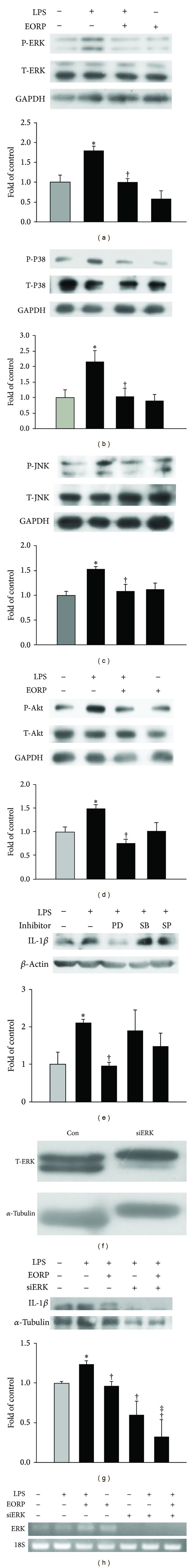
EORP-mediated reduction in LPS-induced IL-1*β* expression is partly dependent on inhibition of phosphorylation of ERK. (a)–(c) Western blot analysis showing the effect of EORP pretreatment on the phosphorylation of (a) ERK1/2, (b) p38, (c) JNK, or (d) Akt in LPS-treated HASMCs. HASMCs were incubated for 24 h with or without 10 **μ**g/mL of EORP, and then the cells were incubated with 10 **μ**g/mL of LPS for 30 min and aliquots of cell lysate containing equal amounts of protein subjected to immunoblotting with the indicated antibodies. (e) Effect of inhibitors of MAPK phosphorylation on IL-1*β* expression in control and LPS-treated HASMCs. HASMCs were incubated for 1 h with 30 **μ**M PD98059 (an ERK1/2 inhibitor), SB203580 (a p38 inhibitor), or SP600125 (a JNK inhibitor) and then for 24 h with or without 10 **μ**g/mL of LPS in the continued presence of the inhibitor and then IL-1*β* expression was measured by Western blotting. (f) ERK-specific siRNA caused a 50% reduction in ERK protein expression by Western blotting. (g) The LPS-induced increase in IL-1*β* expression is inhibited by transfection of HASMCs with ERK1/2-specific siRNA (1 **μ**M). HASMCs were transfected with either control siRNA or ERK1/2-specific siRNA (1 **μ**M) for 48 h then were incubated with 10 **μ**g/mL of EORP for 24 h and then with 10 **μ**g/mL of LPS for 24 h in the continued presence of the same concentration of EORP, and IL-1*β* expression was measured in cell lysates by Western blotting. (h) ERK gene expression under different treatments was analyzed by the RT-PCR assay as described under Section 2. In panels (a)–(e) and (g), the data are expressed as a fold of the control value and are the means ± SEMs (*n* = 3). Total ERK and GAPDH, total p38 and GAPDH, total JNK and GAPDH, total Akt and GAPDH, *β*-actin, and *α*-tubulin were used as the loading control for panels (a), (b), (c), (d), (e), (f), and (g), respectively. *P < 0.05 as compared to the untreated (control) cells. ^†^ *P* < 0.05 as compared to the LPS-treated cells.

The previous studies showed that LPS can activate MAPKs and Akt in the signaling pathway leading to inflammation [26, 28]. In the next set of experiments, the effects of LPS on the activation of the MAPK pathway (ERK1/2, JNK, p38) and Akt, a signaling cascade contributing to IL-1*β* expression, and the effects of EORP or MAPK inhibitors on LPS-stimulated IL-1*β* expression were studied. As shown in Figures 2(a)–2(d), phosphorylation of ERK1/2, p38, JNK, and Akt was significantly increased compared with that of control levels, respectively, at 30 min after addition of LPS. Pretreatment with 10 **μ**g/mL EORP decreased LPS-induced ERK, p38, JNK, and Akt phosphorylation. As shown in Figure 2(e), the increase in IL-1*β* expression in response to LPS treatment was inhibited by 1 h pretreatment with 30 **μ**M PD98059 (an ERK1/2 inhibitor), while SP600125 (a JNK inhibitor) or SB203580 (a p38 inhibitor) had no effect. The inhibitory effect of ERK1/2 was further confirmed by the transfection of ERK siRNA. The effectiveness of the siRNA treatment was validated by showing that ERK-specific siRNA caused a 50% reduction in ERK protein expression (Figure 2(f)). LPS-induced IL-1*β* expression was inhibited by transfection of HASMCs with ERK1/2-specific siRNA (1 **μ**M) (Figure 2(g)). Additional experiments on ERK gene expression showed that it was detected in untreated cells; however, both LPS and EORP treatments did not affect its expression (Figure 2(h)). On the other hand, ERK-specific siRNA treatment decreased ERK gene expression. Both LPS and EORP treatments also did not alter its gene expression. These results indicate that EORP inhibits LPS-induced IL-1*β* expression by preventing LPS-induced phosphorylation of ERK1/2.

### 3.3. EORP Attenuates Phosphorylation and Nuclear Translocation of NF-*κ*B p65 in LPS-Treated HASMCs

**Figure 3:**
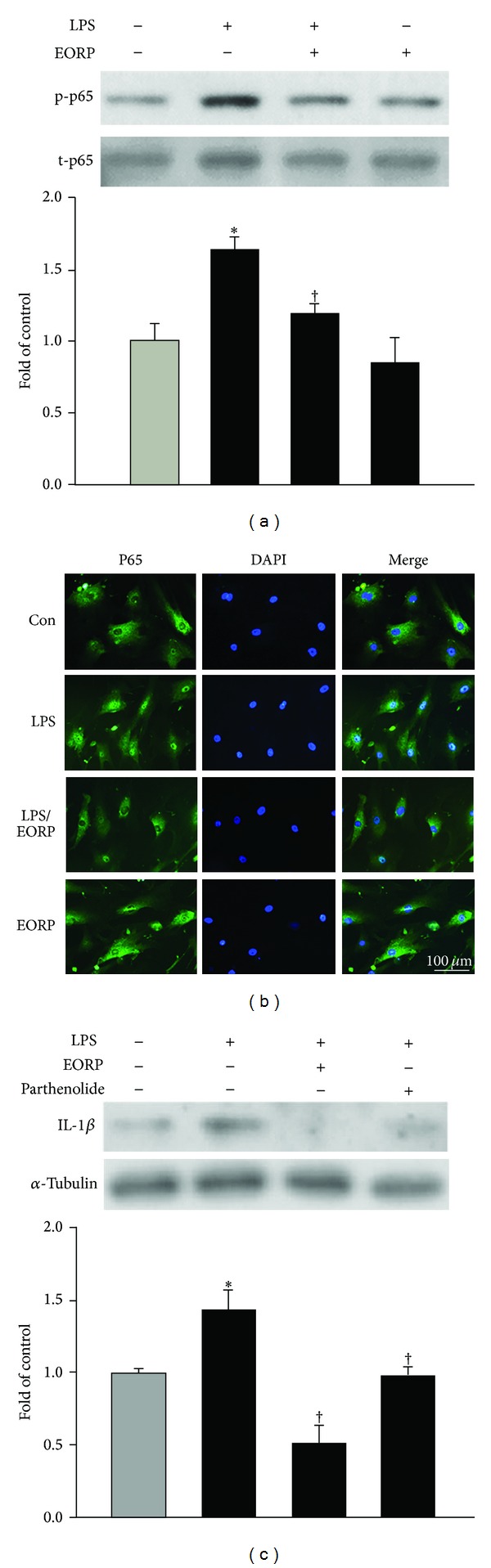
EORP-induced reduction in the upregulation of IL-1*β* expression in LPS-treated HASMCs is mediated by inhibition of both NF-*κ*B p65 phosphorylation and nuclear translocation. (a, b) Western blotting and immunofluorescent staining for NF-*κ*B p65. HASMCs were left untreated or incubated for 24 h with or without 10 **μ**g/mL EORP and then with or without 10 **μ**g/mL of LPS for 90 min in the continued presence of the EORP. (a) The phosphorylation of NF-*κ*B p65 expression is examined by Western blotting. (b) NF-*κ*B p65 expression is indicated by green fluorescence (FITC) and nuclei by blue fluorescence (DAPI). A representative result from three separate experiments is shown. Bar = 100 **μ**m. (c) Cells were incubated for 1 h with 30 **μ**M parthenolide (NF-*κ*B inhibitor) and then coincubated for 24 h with 10 **μ**g/mL of LPS, and then cell lysates were prepared and assayed for IL-1*β* on Western blots. The data are the mean ± SEM (*n* = 3). *P < 0.05 as compared to the untreated (control) cells; ^†^ *P* < 0.05 as compared to the LPS-treated cells.

Transcriptional regulation involving NF-*κ*B activation has been implicated in the stimulator-induced expression of inflammatory cytokines [25]. As shown in Figure 3(a), low basal levels of NF-*κ*B p65 phosphorylation were detected in control cells, and phosphorylation was significantly increased by 90 min treatment with LPS. This increase was markedly inhibited by preincubation with 10 **μ**g/mL EORP for 24 h. To determine whether NF-*κ*B p65 translocation was involved in the pretranslational effects of EORP on IL-1*β* expression, we also studied NF-*κ*B p65 protein levels in the nuclei of LPS-treated HASMCs by immunofluorescent staining. As shown in Figure 3(b), LPS-stimulated HASMCs showed marked NF-*κ*B p65 staining in the nuclei, while EORP-pretreated cells showed weaker nuclear NF-*κ*B expression, but stronger staining in the cytoplasm. Furthermore, the stimulatory effect of LPS on IL-1*β* levels was blocked by coincubation with parthenolide, a NF-*κ*B inhibitor (Figure 3(c)). These results suggest that EORP inhibits LPS-induced IL-1*β* expression by preventing LPS-induced phosphorylation and translocation of NF-*κ*B p65.

### 3.4. EORP Decreases IL-1*β* Protein Expression in Plasma and in Thoracic Aortas of LPS-Injected Mice

**Figure 4:**
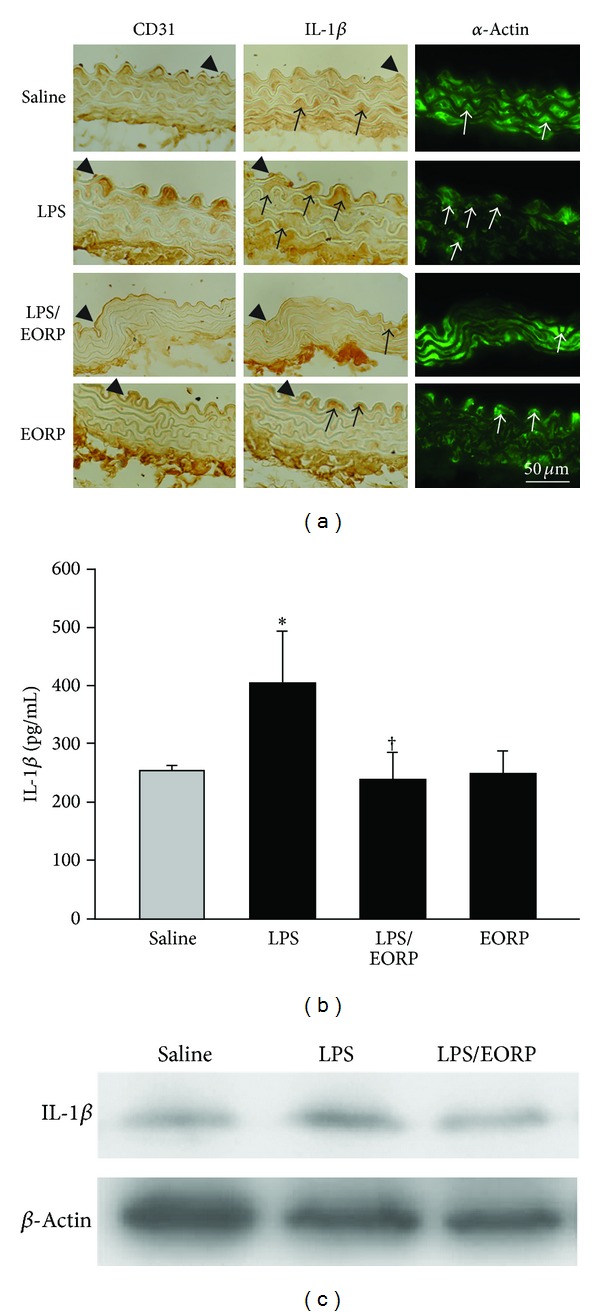
EORPs reduce IL-1*β* expression in LPS-treated mice at the acute inflammatory phase (2 days). (a) Immunohistochemical staining for CD31 (an endothelial cell marker, left panels), IL-1*β* (middle panels), and *α*-actin (a smooth muscle cell marker, right panels) antibodies in serial sections of thoracic aortas. The lumen is uppermost in all sections. The arrowhead and the arrow indicate IL-1*β*-positive overlapping with endothelial cells and smooth muscle cells specific staining, respectively. Bar = 50 **μ**m. (b) IL-1*β* concentration in the plasma was detected by ELISA. The data are the mean ± SEM (*n* = 6). *P < 0.05 as compared to the saline-treated mice (control); ^†^ *P* < 0.05 as compared to the LPS-treated mice. (c) Western blot analysis of IL-1*β* expression in thoracic aortas. *β*-actin was used as the loading control.

**Figure 5:**
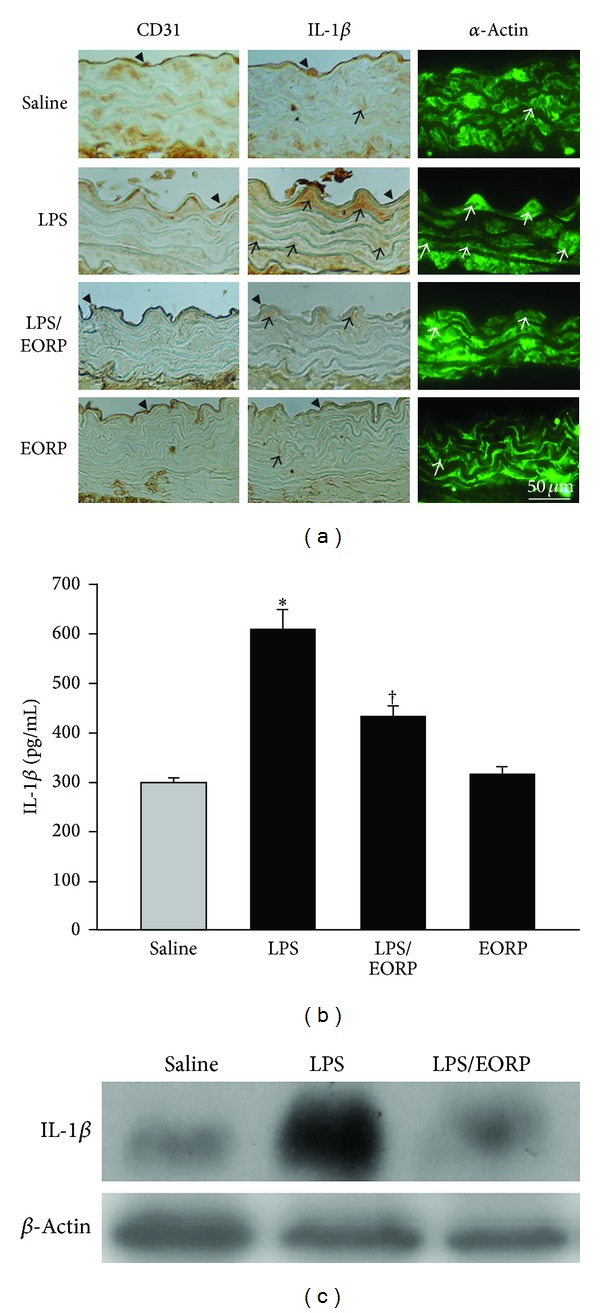
EORPs reduce IL-1*β* expression in LPS-treated mice at the chronic inflammatory phase (2 weeks). (a) Immunohistochemical staining for CD31 (left panels), IL-1*β* (middle panels), and *α*-actin (right panels) antibodies in serial sections on thoracic aortas. The lumen is uppermost in all sections. The arrowhead and the arrow indicate IL-1*β*-positive cells overlapping with endothelial cells and smooth muscle cells specific staining, respectively. Bar = 50 **μ**m. (b) IL-1*β* concentration in the plasma was detected by ELISA. The data are the mean ± SEM (*n* = 6). *P < 0.05 as compared to the saline-treated mice (control); ^†^ *P* < 0.05 as compared to the LPS-treated mice. (c) Western blot analysis of IL-1*β* protein level in thoracic aortas. The expression ratio (IL-1*β*/*β*-actin) was decreased in the EORP (LPS/EORP) group when compared to the LPS-treated group. *β*-actin was used as the loading control.

To determine the effect of EORP on IL-1*β* expression *in vivo*, mice were orally fed with EORP (60 mg/kg/day) prior to treatment with ip injection of LPS (1 mg/kg/day). Serial sections of thoracic aortas were performed to detect IL-1*β* expression and its association with endothelial cells and smooth muscle cells by using CD31 and *α*-actin antibodies as cell markers, respectively. As shown in Figure 4, in the saline- and EORP-treated groups, weak IL-1*β* staining was seen on the vascular wall, while in the LPS-treated group, strong IL-1*β* staining was seen on the vascular walls at the acute inflammatory phase. IL-1*β* expression was associated with endothelial cells and smooth muscle cells. Interestingly, IL-1*β* expression was mainly present in smooth muscle cells of the media rather than in endothelial cells of the intima. In contrast, administration of EORP resulted in weak IL-1*β* staining in the LPS-treated mice. The corresponding levels of IL-1*β* expression were similarly found in plasma and in thoracic aortas by ELISA (Figure 4(b)) and by Western blot (Figure 4(c)), respectively. Furthermore, in the LPS-treated group strong IL-1*β* staining was still seen on the vascular walls at the chronic inflammatory phase as shown in Figure 5. The administration of EORP resulted in weak IL-1*β* staining in the LPS-treated mice. ELISA (Figure 5(b)) showed higher levels of IL-1*β* expression in the LPS-treated group, and EORP treatment notably reduced IL-1*β* expression. The concentration of IL-1*β* in the chronic inflammatory phase was much higher than that in the acute inflammatory phase. Consistent with the *in situ* findings and ELISA, Western blot (Figure 5(c)) showed that IL-1*β* was significantly expressed in the LPS-treated group and EORP treatment reduced the expression.

### 3.5. EORP Regulating LPS-Induced IL-1*β* Expression Was Mediated through the TLR4 Receptor

**Figure 6:**
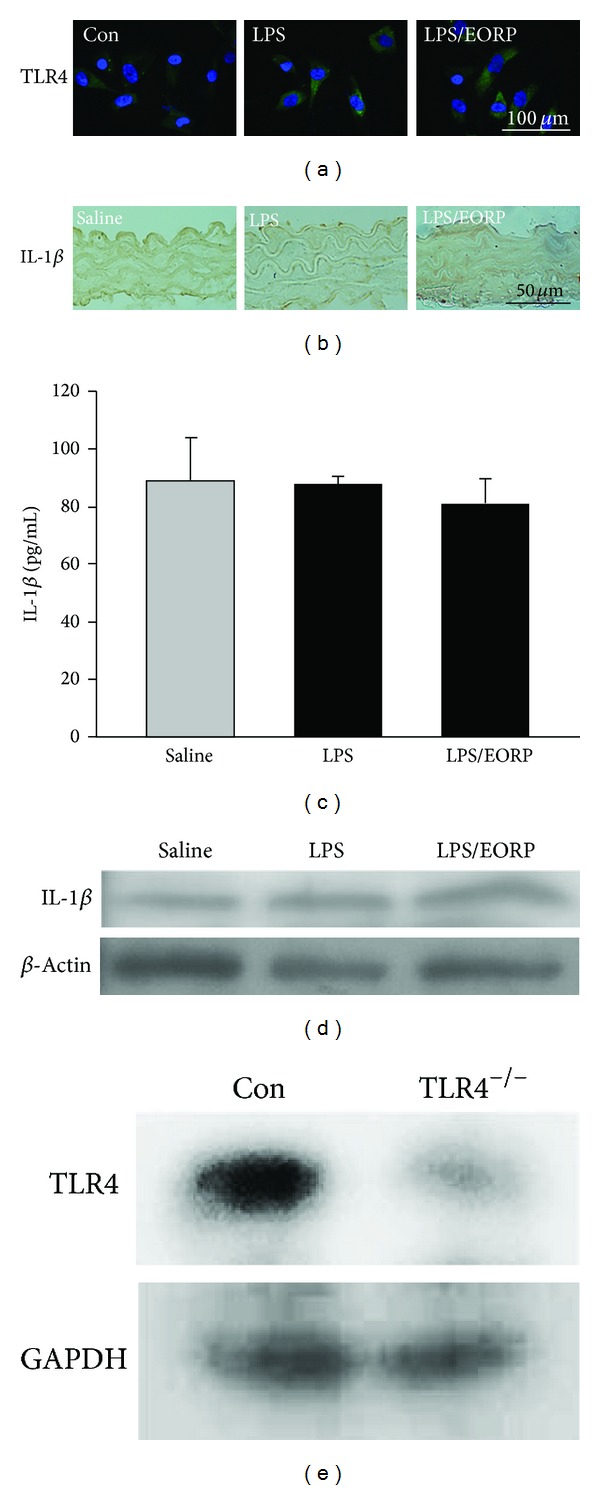
EORP regulating the LPS-induced IL-1*β* expression was mediated through the TLR4 receptor. (a) Immunofluorescent staining for TLR4. HASMCs were left untreated or incubated for 24 h with or without 10 **μ**g/mL EORP and then incubated with the 10 **μ**g/mL LPS for another 24 h. TLR4 expression was indicated by green fluorescence (FITC) and nuclei by blue fluorescence (DAPI). The untreated HASMCs were used as the control cells. Bar = 100 **μ**m. (b) Immunohistochemical staining for IL-1*β* expression in thoracic aortas of TLR4^−/−^ mice. The lumen is uppermost in all sections. Bar = 50 **μ**m. (c) IL-1*β* concentration in plasma was detected by ELISA. The data are the mean ± SEM (*n* = 5). (d) Western blot analysis of IL-1*β* in thoracic aortas of TLR4^−/−^ mice. The saline-treated TLR4^−/−^ mice were used as the control group in (b), (c), and (d). *β*-actin was used as the loading control. (e) Western blot analysis of TLR4 expression in thoracic aortas of C57BL6J (con) and TLR4^−/−^ mice. GAPDH was used as the loading control. F: CAGACCATGATCACACAGGG R: TGGAAAGATGGGCCTGTTAG.

The effects of LPS or EORP on the cytokine expression via TLR4-modulated protein kinase signaling pathways were studied by immunofluorescent microscopy. In untreated cells, TLR4 expression was weak (Figure 6(a)), whereas cells treated for 24 h with LPS showed strong TLR4 expression and this effect was reduced by pretreatment with EORP. To elucidate whether EORPs modulate LPS-induced IL-1*β* expression through the TLR4 receptor, TLR4^−/−^ mice were used in the present study. As shown in Figures 6(b)–6(d), the level of IL-1*β* expression in LPS-treated or in LPS/EORP-treated group was very low and was similar to that of the saline-treated group examined by immunofluorescent staining, ELISA, and Western blot, respectively. The expression of TLR4 on TLR4^−/−^ mice was confirmed by Western blot (Figure 6(e)). Based on these findings, the LPS-induced IL-1*β* expression was mainly mediated through the activation of TLR4 receptor.

## 4. Discussion

Herein, we demonstrated that EORP treatment effectively blocked IL-1*β* expression both *in vitro* in LPS-stimulated HASMCs and *in vivo* in thoracic aortas of LPS-treated mice. EORP decreased IL-1*β* expression in LPS-treated HASMCs, and the effect might be mediated through inhibition of ERK phosphorylation, NF-*κ*B activation, and TLR4 receptor pathway.

Reishi extract was chosen for testing, as it has long been known as a healthy food and used as traditional Chinese medicines. Its beneficial effects are thought to be due to its anti-inflammatory, antitumor, antioxidant, and immunomodulatory actions [16–18]. A *Ganoderma* extract prevented albumin-induced oxidative damage of proximal tubular epithelial cells in an experimental setting, mimicking the proteinuric state [24], and reduced LPS-induced superoxide anion production by macrophages [29]. The triterpene extract from *G. lucidum* (GLT) suppressed the inflammatory response *in vitro* and *in vivo* by downregulating the expression of inducible nitric oxide synthase (iNOS) and cyclooxygenase 2 (COX-2) and the production of TNF-*α* and IL-6 in LPS-induced endotoxemic mice [22]. *Ganoderma lucidum* extracts inhibited the production of microglia-derived proinflammatory and cytotoxic factors, including nitric oxide, TNF-*α*, and IL-1*β* in activated microglia [23]. *G. lucidum* polysaccharide-linked peptide reduced the production of proinflammatory cytokines (interleukin-6 and monocyte chemoattractant protein-1) by activated rheumatoid synovial fibroblasts [30]. Our previous report demonstrated an EORP-associated protective mechanism against bacteria infection involving the clearance of LPS by macrophages [31]. We also demonstrated that EORP attenuates endotoxin-induced ICAM-1 expression in cultured smooth muscle cells and in the neointima in mice [27]. Moreover, EORP prevented PDGF-stimulated smooth muscle cell proliferation *in vitro* and neointimal hyperplasia in the endothelial-denuded artery *in vivo* [32]. The present study is the first to report that EORP strongly reduces the expression of IL-1*β* protein in LPS-treated HASMCs.

The activation of various intracellular pathways by inflammatory stimuli, such as LPS, is required for the production of these adhesion molecules and proinflammatory chemokines [28]. LPS-induced inflammatory responses, such as ICAM-1 expression in HASMCs, are regulated via TLR4 expression [33]. Most notably, the TLR4-mediated signaling pathway for LPS, leading to the activation of various intracellular kinases, including MAPKs and transcription factors, appears to be critical for the development of vascular inflammation and diseases [26]. Our study showed that LPS caused strong activation of three MAPK subtypes in HASMCs, as reported in a previous study [34]. However, the involvement of their activation in the protective mechanism of EORP remains unclear. In the present study, the increase in IL-1*β* expression induced by LPS was markedly suppressed in the presence of an ERK inhibitor (PD98059) but not a p38 inhibitor (SB203580) or a JNK inhibitor (SP600125). IL-1*β* expression was also inhibited by ERK-specific siRNA. EORP decreased LPS-induced ERK phosphorylation. Thus, one of the mechanisms by which EORP reduces LPS-induced IL-1*β* expression involves a reduction in ERK1/2 activation. Consistent with our results, a *G. lucidum* extract inhibited the oxidative stress-induced phosphorylation of ERK1/2 in breast cancer cells, resulting in suppression of IL-8 secretion and finally in inhibition of cell migration [35]. Another study showed that a *G. lucidum* extract inhibited prostate cancer-dependent angiogenesis by inhibition of phosphorylation of ERK1/2 and Akt kinases [36]. EORP has been shown to inhibit LPS-induced inflammatory cytokine in murine RAW264.7 cells by suppression of the phosphorylation of ERK1/2 and JNK [22]. Active lipids of *Ganoderma lucidum* spores are able to enhance apoptosis in THP-1 cells through inhibition of ERK1/2 and Akt and activation of JNK1/2 signaling pathways [32], whereas triterpenes from *Ganoderma lucidum* induce autophagy in colon cancer through the inhibition of P38 MAPK [37]. Our previous studies showed that EORP reduces LPS-induced the ICAM-1 expression by the decrease of ERK1/2 activation [27], and prevents the PDGF-stimulated HASMCs proliferation through inhibition of JNK activation [32]. The differences between the above results in terms of the pathways involved may be related to the different cell types used and the cytokines examined.

NF-*κ*B is one of the most ubiquitous transcription factors and regulates the genes involved in cellular proliferation, inflammatory responses, and cell adhesion [25]. NF-*κ*B transcriptional activity can be modulated by the phosphorylation of MAPKs. LPS-induced IL-1*β*, TNF-*α*, IL-6, COX-2, and iNOS in Raw264.7 macrophages via the NF-*κ*B activation [38]. These findings raised the possibility that EORP reduces IL-1*β* expression through a reduction in NF-*κ*B activity. Our study demonstrated that the EORP-induced decrease in IL-1*β* expression was mediated through inactivation of NF-*κ*B binding activity. Pretreatment with an NF-*κ*B inhibitor also suppressed the LPS-induced increase in IL-1*β* expression. This is consistent with a previous report that a *G. lucidum* extract inhibited the proliferation of human breast cancer cells by downregulation of NF-*κ*B signaling [39]. *Ganoderma lucidum* polysaccharide peptide (GL-PP) reduced the production of proinflammatory cytokines in activated rheumatoid synovial fibroblast by inhibiting the NF-*κ*B transcription pathway [30]. Another study also showed that a *Ganoderma lucidum* polysaccharides (Gl-PS) prevented pancreatic islets from alloxan-induced damage by inhibiting activation of NF-*κ*B [40]. NF-*κ*B is activated by signals possibly involving phosphorylation of the I*κ*B subunit and its dissociation from the inactive cytoplasmic complex, followed by translocation of the active p50/p65 dimer to the nucleus [41]. We demonstrated that the EORP-induced decrease in IL-1*β* expression was mediated through inhibition of NF-*κ*B p65 phosphorylation and translocation.

LPS-induced systemic inflammatory responses increase neointimal formation after balloon injury and stent implantation, and inflammatory cytokines are produced by VSMCs in the neointima [42]. In the present study, EORP was shown to significantly reduce IL-1*β* expression in thoracic aortas in LPS-treated mice. On the basis of the probable involvement of IL-1*β* expression in migration and proliferation of SMCs, our findings suggest an additional mechanism by which EORP treatment may be important in preventing the progression of cardiovascular disorders and inflammation. It has been well known that the LPS induces the production of proinflammatory molecules through TLR4-activated signaling pathway, and IL-1 was the one of proinflammatory molecules [43]. Another study also showed that IL-1*β* expression is mediated by both TLR4 and Nod1 pathways in the cultured HAPI cells stimulated by LPS [44]. In addition, LPS-induced TLR4 protein expression and mRNA stabilization in HASMCs are mediated by NADPH oxidase-related ROS production and MAPK signaling pathways [45]. Consistent with the previous studies, LPS significantly induced TLR4 expression in HASMCs. Moreover, LPS did not affect IL-1*β* expression in thoracic aortas in TLR4^−/−^ mice. There was no difference of IL-1*β* expression in all groups of TLR4^−/−^ mice by using immunohistochemistry, cytokine ELISA, and Western blot. Based on these findings, we suggested that EORP suppressed the LPS-induced IL-1 expression through inhibition of TLR4 activation.

In conclusion, this study provides the first evidence that EORP reduces IL-1*β* expression in LPS-treated HAMSCs both *in vitro* and *in vivo*. The present data suggest that these effects might be mediated through inhibition of ERK phosphorylation and NF-*κ*B activation. The results of the present study suggest a possible therapeutic role for *G. lucidum* extract in cardiovascular disorders and in inflammatory diseases.
